# Nonlinear effects of ambient temperature and treatment days on nocturnal enuresis using generalized additive models

**DOI:** 10.1038/s41598-025-28240-x

**Published:** 2025-12-29

**Authors:** Teruo Miyano, Takahiro Arai, Yuta Onuki, Tomoko O. Morita, Hirokazu Ikeda

**Affiliations:** 1 Japan Data Science Research Institute, 3F Kato Building, 1-9-18 Hiroo, Shibuya-ku, Tokyo 150-0012 Japan; 2 Graduate School of Health Management, Keio University, Fujisawa, Kanagawa 252-0883 Japan; 3 School of Management and Information Sciences, Tama University, Tama, Tokyo 206-0022 Japan; 4 Children’s Center, Showa Medical University Northern Yokohama Hospital, Yokohama, Kanagawa 224-8503 Japan

**Keywords:** Weather conditions, Treatment days, Children, Bed-wetting, Medical research, Epidemiology, Outcomes research, Paediatric research, Diseases, Kidney diseases, Urogenital diseases, Health care, Paediatrics

## Abstract

To examine the effects of the average daily ambient temperature and treatment days on the outcomes of patients with nocturnal enuresis (NE), a common urinary symptom in children. The panel data of 19 children with NE (12 boys and 7 girls; median age: 9 years; age range: 6–15) were prospectively collected (up to 1,469 days; total number of records: 3194). NE status (presence of incontinence), treatment type, and weather-related variables (ambient temperature, precipitation, sunshine hours, and barometric pressure) were recorded. Factor analyses were subsequently performed using a generalized additive model (GAM). Among the patient features, treatment type, and weather-related variables, the average daily ambient temperature and treatment days were identified as risk factors for NE. Specifically, NE risk increased with an average daily ambient temperature of < 16.7 °C and decreased with a daily average ambient temperature of > 20.1 °C. An inverse correlation between NE risk and treatment days was observed in all cases. Notably, long-term treatment of approximately ≥ 1465 days significantly reduced the NE risk, despite repeated exacerbations. The average daily ambient temperature and treatment days affect the risk of NE.

**Supplementary Information:**

The online version contains supplementary material available at 10.1038/s41598-025-28240-x.

## Introduction

Nocturnal enuresis (NE) is a common condition in children that can cause significant long-term psychosocial risks if left untreated, despite being benign from a physical standpoint^1^. According to the International Children’s Continence Society (ICCS), NE, defined as involuntary urination during sleep in children aged ≥ 5 years, is classified into the primary and secondary forms, as well as monosymptomatic (without other lower urinary tract symptoms) and non-monosymptomatic NE (with additional urinary symptoms)^2^. Primary etiological factors of primary NE include nocturnal polyuria, reduced bladder capacity, detrusor overactivity, impaired arousal mechanisms, psychological stress, and genetic predisposition, with an intricately multifactorial pathophysiology^3,4^. Furthermore, seasons influence NE, with symptoms known to worsen particularly during winter. Tas et al.^5^ reported a significantly higher rate of NE episodes during winter than during summer, while Murillo et al.^6^ identified an increased failure rate of desmopressin treatment during the winter months. These findings suggest that low temperatures may contribute to decreased antidiuretic hormone secretion, reduced bladder capacity, and increased bladder contractions. However, to the best of our knowledge, specific meteorological factors such as ambient temperature, atmospheric pressure, and humidity have been under-investigated in relation to NE. Therefore, this study focused on the meteorological variables and conducted an exploratory evaluation of their impact on primary NE.

## Methods

This study was duly approved by the Ethics Committee of Showa Medical University Hospital (approval number: 22–161-B), and written informed consent was obtained from their parents/legal guardians. All procedures were conducted in compliance with relevant Japanese guidelines and regulations. In this study, we mainly included patients diagnosed with primary NE, according to the diagnostic criteria established by the International Children’s Continence Society (ICCS). Pediatric patients with no evidence of organic or physiological abnormalities in the urinary tract or central nervous system were enrolled (detailed inclusion criteria are provided in the Supplemental Data). The study was conducted from December 15, 2022, to February 1, 2024.Throughout this period, participants, along with their parents or legal guardians were instructed to maintain voiding diaries to systematically document daily urination frequency and episodes of urinary incontinence.

Additionally, clinically relevant patient information pertinent to the assessment of primary NE was collected, including sex, age, residential area, height, weight, and treatment modalities such as desmopressin (DDAVP), solifenacin succinate, vibegron, alarm therapy, and osmotic laxatives. To evaluate meteorological variables, six parameters—daily mean temperature, diurnal temperature range, total precipitation, sunlight days, average atmospheric pressure, and average humidity—were extracted from publicly available data provided by the Japan Meteorological Agency (Supplementary Table 1)^7^.

To assess the association between meteorological variables and NE, we constructed and applied a generalized additive model (GAM), which incorporated all meteorological variables and patient information. The significance of spline terms (nonlinear terms) within this model was evaluated, and statistically significant variables were included in the final model. Consequently, the daily mean ambient temperature (*p* = 0.01) and treatment days (*p* < 0.001) were incorporated into the final model. To address interindividual variability, individual dummy variables were additionally included. Statistical analyses were performed using R version 4.3.3 (R Core Team), and the “mgcv” package was used for the GAM analysis. Default spline settings were applied for model fitting and smoothing effect evaluation. All tests were two-sided, and* p*-values < 0.05 were considered statistically significant.

## Results

Nineteen patients with only primary NE were enrolled in this study, excluding the ones with secondary enuresis. Both monosymptomatic and non-monosymptomatic cases, as well as treatment resistant and newly diagnosed ones, were included in the analysis without further distinction. Unbalanced panel data from 3,222 daily records were prospectively generated, with 3,194 valid ones from the enrolled 19 individuals. The range of treatment days was broad (1–1,469 days), enabling the evaluation of long-term therapeutic effects. The characteristics of the patients enrolled in this study are described in Table 1. Analysis of the risk factors for NE using the GAM analysis indicated that standard treatment with DDAVP (odds ratio [OR]: 0.33, 95% confidence interval [CI] 0.16, 0.69), vibegron (beta3-adrenergic receptor agonist) (OR 0.17, 95% CI 0.07, 0.43), and alarm therapy (OR 0.33, 95% CI 0.15, 0.70) significantly mitigated the risk of NE (Table 2). Conversely, solifenacin succinate (a muscarinic receptor antagonist) (OR 1.82, 95% CI 0.83, 4.00) and osmotic laxatives (OR 1.10, 95% CI 0.32, 3.82) did not significantly reduce NE risk.

**Table 1:** Patient characteristics.

Characteristic	Overall, n = 19
Age	
Mean±SD	9.6±2.5
Median	9
Range	(6–15)
Sex	
Male	12
Female	7
Height (cm)	
Mean±SD	128.2±11.1
Median	127.2
Range	111.0–147.7
Weight (kg)	
Mean±SD	27.2±6.7
Median	27.6
Range	17.5–40.0
Baseline NE severity^*1^ (number of wet nights/month)	
Mean	9.2
Number of treatments	
Vasopressin (DDAVP)	11
Solifenacin succinate	9
Vibegron	6
Alarm therapy	10
Osmotic laxatives	4
Duration of drug administration (%)	
Vasopressin (DDAVP)	49.7
Solifenacin succinate	46.4
Vibegron	12.5
Osmotic laxatives	17.0
Frequency of drug administration	
Vasopressin (DDAVP)	120–240 μg/day
Solifenacin succinate	2.5–5.0 mg/day
Vibegron	25–50 mg/day
Osmotic laxatives^*2^	
Magnesium oxide	0.02–0.10 g/kg/day
Polyethylene glycol	1–3 packets/day

*^1^Calculations were made assuming 30-day months.

*^2^Osmotic laxatives were used to relieve bladder pressure caused by constipation.

**Table 2:** Odds Ratios of Variables and Adjusted R-Squared in the Generalized Additive.

Category	Variables	Model (R-squared = 0.38)			
			95% CI		
		OR	Lower	Upper	*p*-value
Intervention	Vasopressin (DDAVP)	0.33	0.16	0.69	0.00
	Solifenacin succinate	1.82	0.83	4.00	0.14
	Vibegron	0.17	0.07	0.43	0.00
	Alarm therapy	0.33	0.15	0.70	0.00
	Osmotic laxatives	1.10	0.32	3.82	0.88
	Case Number 1 (Reference)	─	─	─	–
Individual dummy	Case Number 2	0.34	0.00	118.48	0.72
	Case Number 3	0.03	0.00	9.67	0.24
	Case Number 4	1.38	0.00	405.64	0.91
	Case Number 5	0.17	0.00	51.87	0.54
	Case Number 6	0.13	0.00	58.33	0.52
	Case Number 7	0.26	0.04	1.86	0.18
	Case Number 8	1.15	0.60	2.17	0.68
	Case Number 9	2.40	0.00	1302.46	0.78
	Case Number 10	2.14	0.49	9.31	0.31
	Case Number 11	0.13	0.00	66.78	0.52
	Case Number 12	0.11	0.00	39.15	0.46
	Case Number 13	0.01	0.00	2.67	0.10
	Case Number 14	0.01	0.00	4.18	0.14
	Case Number 15	2.29	0.01	825.74	0.78
	Case Number 16	0.00	0.00	0.43	0.02
	Case Number 17	0.03	0.00	7.34	0.20
	Case Number 18	0.02	0.00	7.79	0.20
	Case Number 19	0.27	0.00	119.47	0.67

The dummy variables for the cases are referenced from Case 1.

Abbreviations: OR, odds ratio; CI, confidence interval.

**Fig. 1:**
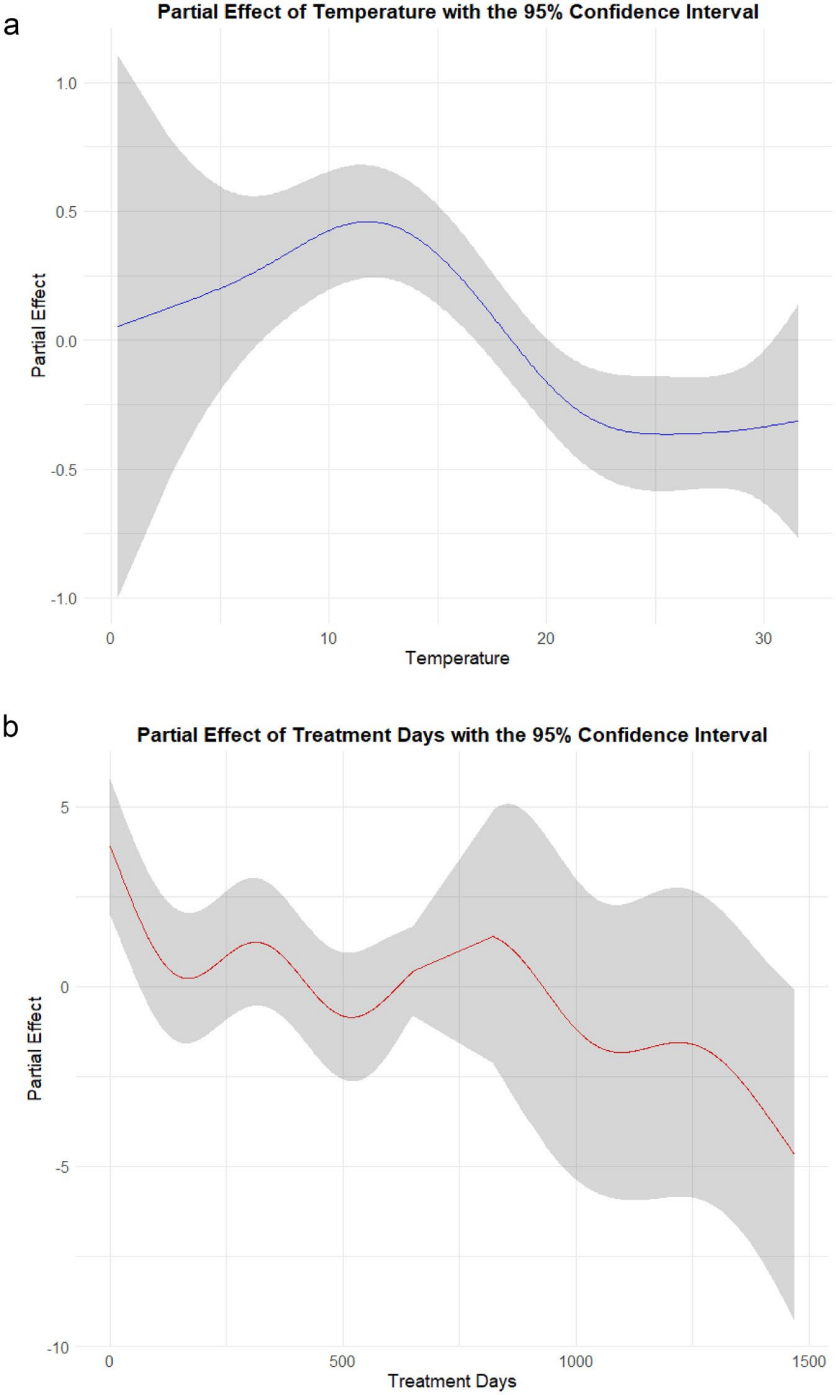
Partial effect of the: (**a**) average daily ambient temperature; (**b**) treatment days on nocturnal enuresis symptoms. The solid lines indicate the predicted value, while gray areas indicate the 95% confidence intervals.

According to the GAM analysis, the smoothing terms for the average ambient temperature and treatment days were statistically significant (average ambient temperature, p = 0.001; treatment days, p < 0.001). Moreover, these terms significantly influenced the overall outcomes (Table 3). Based on the partial effect plots (log odds), lower (< 16.7 °C) or higher ambient temperatures (> 20.1 °C) were associated with an increased risk of incontinence with NE (Fig. 1a). Furthermore, the treatment days was shown to be inversely correlated with the risk of incontinence (Fig. 1b).

**Table 3:** Summary of smoothing term results from the Generalized Additive Model (GAM).

Smoothing term	edf	Ref.df	Chi.sq	*p*-value
Average ambient temperature	3.82	4.77	20.05	0.001 *
Treatment duration (Days)	8.36	8.86	127.40	< 0.001 *

Abbreviations: S, the smoothing term; edf, the estimated degree of freedom; Ref.df, the reference degree of freedom; and Chi.sq, the chi-square statistic used to test the significance of the smoothing term. *Statistically significant at the 5% level.

The smooth term for the average ambient temperature was statistically significant (estimated degree of freedom = 3.82,* p* = 0.007), suggesting a nonlinear association between daily average ambient temperature and NE risk.

The effect of treatment days on outcomes was nonlinear, with repeated exacerbations and improvements. A tendency for improvement was observed in the early stages of treatment, which was subsequently followed by a trend toward worsening from day 65, and then a renewed trend toward improvement noted after 1,463 days of treatment. The 95% CIs for the partial effect plots used to assess the effect of treatment days on NE risk were wide, with the majority of the estimates crossing zero. The effect of the average ambient temperature on NE was smaller than that of the days of treatment.

## Discussion

The strengths of this study include (1) identification of a correlation between the mean ambient outdoor temperature and worsening of NE; (2) the ability to capture the longitudinal course of NE over an extended 1,463-day period; and (3) the observed significant association between prolonged treatment days and improvement in NE symptoms.

Consistent with existing studies suggesting the aggravation of NE symptoms during winter, our findings revealed that low mean and extremely high ambient temperatures constitute a significant risk factor for NE exacerbation.

Several mechanisms may explain the association between low ambient temperatures and NE deterioration. For example, Mazumder et al. reported an inverse correlation between ambient temperature and 24-h urine volume^8^. Tas et al. observed that low temperatures are associated with reduced antidiuretic hormone secretion and diminished bladder capacity, resulting in increased urinary frequency^5^. Altogether, it indicates that cold ambient temperatures affect thermoregulation and urine production in children, thereby exacerbating NE. Additionally, peripheral vasoconstriction induced by cold stimuli may increase renal blood flow and urine output, further deteriorating NE.

Conversely, in cases of high ambient temperatures, increased water intake is a plausible contributor. Bergel et al. demonstrated that a 1 °C rise in temperature is associated with an increase in daily water consumption from 0.41 to 3.20 L^9^. Moreover, air conditioning use during hot periods reduces fluid loss via perspiration, which may elevate urine volume.

A nonlinear positive correlation was observed between treatment days and symptom improvement in NE. The deterioration of transient symptom observed around day 65 after treatment initiation could be an initial improvement due to high motivation and good treatment adherence, which tend to decline around day 65, leading to the exacerbation of the temporary symptoms. We speculate that this change in motivation, together with seasonal fluctuations in enuresis symptoms, contributes to the observed nonlinearity. The improvement in symptoms observed near day 1463 presumably reflects the maturation of bladder function with age. This extended longitudinal follow-up suggests that symptomatic NE cycles of exacerbation and remission are influenced by the environment fluctuations, which ultimately improves with bladder maturation.

In present study, employing the GAM enabled the analysis of nonlinear effects of the mean temperature and treatment days on NE risk, revealing complex relationships often masked by traditional linear models. The long-term follow-up of pediatric NE cases unraveled the rare and clinically valuable data. However, considering this is an exploratory study, confirmatory trials are warranted to validate these findings.

The limitations of this study include the relatively small sample, which limits the generalizability of its findings, and inadequate assessment of confounding variables such as fluid intake, stress, and sleep quality. Future confirmatory studies must involve larger cohorts with a more comprehensive evaluation of potential confounders.

In conclusion, the mean ambient temperature and treatment days exert nonlinear influences on NE risk, highlighting the importance of environmental factors and long-term treatment assessment in management strategies. These findings further elucidate NE pathophysiology and support the development of effective therapeutic options.

## Supplementary Information

Supplementary Information 1.

Supplementary Information 2.

Supplementary Information 3.

Supplementary Information 4.

## Data Availability

The datasets analyzed during the current study are not publicly available because we do not have individual consent to release the data; however, they are available from the corresponding author on reasonable request.

## Abbreviations

CI: Confidence interval
GAM: Generalized additive model
NE: Nocturnal enuresis
OR: Odds ratio
