# Supplementary material for: Nonlinear effects of ambient temperature and treatment days on nocturnal enuresis using generalized additive models
Source: Sci Rep. 2025 Dec 29;15:44719. doi: 10.1038/s41598-025-28240-x (PMC12748616; doi:10.1038/s41598-025-28240-x)
Supplement: Supplementary file 1 — Supplementary Information 1. [file 41598_2025_28240_MOESM1_ESM.docx]

**Supplementary Data**

**Study Population**

Prospective approval of protocol deviations to recruitment and enrollment criteria, also known as protocol waivers or exemptions, is not permitted.

**1. Inclusion criteria**

1. Patients with nocturnal enuresis who are either admitted to or attending the Pediatric Center at Showa University Northern Yokohama Hospital or the Department of Pediatrics at Showa University Fujigaoka Hospital.
2. Patients diagnosed with nocturnal enuresis based on the diagnostic criteria of the International Consensus on Continence Disorders in Children and Adolescents (ICCS) and who have been found to have nocturnal enuresis at least once a month.
3. Patients who are either filling in a nocturnal enuresis diary every day or using an alarm device (Piscall) to upload the date of their nocturnal enuresis to the cloud.
4. Patients who have obtained written consent from a substitute decision-maker for participation in the study.

**2. Exclusion criteria**

1. Patients who did not give written consent for this study
2. Patients with neurodevelopmental disorders such as autism or ADHD
3. Patients with concomitant organic diseases of the renal and urinary tract such as neurogenic bladder
